# Supplementary material for: Bayesian refinement of protein structures and ensembles against SAXS data using molecular dynamics
Source: PLoS Comput Biol. 2017 Oct 18;13(10):e1005800. doi: 10.1371/journal.pcbi.1005800 (PMC5662244; doi:10.1371/journal.pcbi.1005800)
Supplement: S1 Archive — (BZ2) [file pcbi.1005800.s011.tar.bz2 › gromacs_4.62_bayesian_saxs_refi/share/html/online/protonate.html]

protonate


| protonate Main Table of Contents |

**VERSION 4.6  
Sat 19 Jan 2013**

---

### Description

protonate reads (a) conformation(s) and adds all missing
hydrogens as defined in ffgmx2.hdb. If only -s is
specified, this conformation will be protonated, if also -f
is specified, the conformation(s) will be read from this file
which can be either a single conformation or a trajectory.

If a pdb file is supplied, residue names might not correspond to
to the GROMACS naming conventions, in which case these residues will
probably not be properly protonated.

If an index file is specified, please note that the atom numbers
should correspond to the **protonated** state.

### Files

| option | filename | type | description |
| --- | --- | --- | --- |
| **-s** | topol.tpr | Input | Structure+mass(db): tpr tpb tpa gro g96 pdb |
| **-f** | traj.xtc | Input, Opt. | Trajectory: xtc trr trj gro g96 pdb cpt |
| **-n** | index.ndx | Input, Opt. | Index file |
| **-o** | protonated.xtc | Output | Trajectory: xtc trr trj gro g96 pdb |

### Other options

| option | type | default | description |
| --- | --- | --- | --- |
| **-[no]h** | bool | no | Print help info and quit || **-nice** | int | 0 | Set the nicelevel || **-b** | time | 0 | First frame (ps) to read from trajectory || **-e** | time | 0 | Last frame (ps) to read from trajectory || **-dt** | time | 0 | Only use frame when t MOD dt = first time (ps) |

---

http://www.gromacs.org  
gromacs@gromacs.org
